# The Garlic Compound, Diallyl Trisulfide, Attenuates Benzo[a]Pyrene-Induced Precancerous Effect through Its Antioxidant Effect, AhR Inhibition, and Increased DNA Repair in Human Breast Epithelial Cells

**DOI:** 10.3390/nu16020300

**Published:** 2024-01-19

**Authors:** Dominique T. Ferguson, Equar Taka, Samia Messeha, Hernan Flores-Rozas, Sarah L. Reed, Bryan V. Redmond, Karam F. A. Soliman, Konan J. W. Kanga, Selina F. Darling-Reed

**Affiliations:** 1 Pharmaceutical Sciences Division, College of Pharmacy and Pharmaceutical Sciences, Florida A&M University, Tallahassee, FL 32307, USA; dominique3.ferguson@famu.edu (D.T.F.); equar.taka@famu.edu (E.T.); samia.messeha@famu.edu (S.M.); hernan.floresrozas@famu.edu (H.F.-R.); sarah1.reed@famu.edu (S.L.R.); karam.soliman@famu.edu (K.F.A.S.); 2 Department of Neuroscience, University of Rochester Medical Center, Rochester, NY 14642, USA; bryan_redmond@urmc.rochester.edu; 3 Department of Biomedical Sciences, College of Medicine, Florida State University, Tallahassee, FL 32306, USA; kwk06@fsu.edu

**Keywords:** *Allium sativum*, diallyl trisulfide, benzo[a]pyrene, reactive oxygen species, antioxidants, carcinogenesis

## Abstract

Exposure to B[a]P, the most characterized polycyclic aromatic hydrocarbon, significantly increases breast cancer risk. Our lab has previously reported that diallyl trisulfide (DATS), a garlic organosulfur compound (OSC) with chemopreventive and cell cycle arrest properties, reduces lipid peroxides and DNA damage in normal breast epithelial (MCF-10A) cells. In this study, we evaluated the ability of DATS to block the B[a]P-induced initiation of carcinogenesis in MCF-10A cells by examining changes in proliferation, clonogenic formation, reactive oxygen species (ROS) formation, 8-hydroxy-2-deoxyguanosine (8-OHdG) levels, and protein expression of ARNT/HIF-1β, CYP1A1, and DNA POLβ. The study results indicate that B[a]P increased proliferation, clonogenic formation, ROS formation, and 8-OHdG levels, as well as increasing the protein expression of ARNT/HIF-1β and CYP1A1 compared to the control. Conversely, DATS/B[a]P co-treatment (CoTx) inhibited cell proliferation, clonogenic formation, ROS formation, and 8-OHdG levels compared to B[a]P alone. Treatment with DATS significantly inhibited (*p* < 0.0001) AhR expression, implicated in the development and progression of breast cancer. The CoTx also attenuated all the above-mentioned B[a]P-induced changes in protein expression. At the same time, it increased DNA POLβ protein expression, which indicates increased DNA repair, thus causing a chemopreventive effect. These results provide evidence for the chemopreventive effects of DATS in breast cancer prevention.

## 1. Introduction

For centuries, garlic (*Allium sativum*) has been used as a prophylactic and therapeutic medicinal agent for treating and preventing disease [1]. As a functional and medicinal food, garlic has been shown to have antioxidant, antitumorigenic, anti-inflammatory, and glucose- and cholesterol-lowering properties [2,3]. It is also implicated in the reduction of carcinogen-induced DNA strand breaks, induced cell cycle arrest and apoptosis, inhibited RNA and DNA synthesis, and suppressed angiogenesis [4]. Alliinase catalyzes alliin to allicin once the garlic is chopped, ground, or pressed. Allicin is an unstable compound that easily converts into sulfur-containing organosulfur compounds (OSCs). The OSCs converted from allicin are composed of diallyl sulfide (DAS), diallyl disulfide (DADS), and diallyl trisulfide (DATS). The anti-carcinogenic effects of garlic are attributed to OSCs [4].

Diallyl trisulfide (DATS) has greater anticancer effects than garlic’s water-soluble sulfur compounds due to its structure’s increased number of sulfur atoms [5]. DATS was shown to have a variety of pharmacological and biological activities, including antitumor, antioxidant, antimicrobial, and anti-inflammatory actions [1]. The inhibitory effects of DATS on tumor growth involve various mechanisms such as inhibition of ROS, cell proliferation, cell cycle arrest, and tumor cell migration and invasion [1]. These properties reinforce DATS’s potential as a chemopreventive or chemotherapeutic agent.

In situ and invasive breast cancer incidences are higher in urban areas than in rural areas in the United States, regardless of race [6]. Epidemiological studies have implied that a positive association exists between greater urbanization, higher in situ breast cancer incidence, and invasive incidences in women [7]. Benzo[a]pyrene (B[a]P) is a classified group 1 complete human carcinogen found extensively in low urban areas [8,9]. B[a]P, the most characterized component of the polycyclic aromatic hydrocarbon (PAH) family, is a known environmental contaminant and toxicant formed from incomplete combustion of organic materials [10]. B[a]P exposure can be through ambient air, smoke, car exhausts, residential and commercial heating, biomasses, and indoor/outdoor sources. Reactive metabolites of B[a]P produce mutagenesis via DNA adducts and strand breaks, leading to cancer [11]. The long-term effect of cumulative airborne B[a]P exposure is significantly associated with breast cancer risk and increased incidence of sarcomas, liver, and lung tumors [12].

Our lab has previously reported that the chemopreventive/chemotherapeutic potential of the garlic OSC, DATS, lies in its ability to induce apoptosis and cell cycle arrest in breast cancer cells and other cancers while attenuating S-phase cell cycle transition, suppressing ROS production and inhibiting DNA damage in breast epithelial (MCF-10A) cells [13,14]. Previous studies have demonstrated that DATS can induce apoptosis through oxidative modification and inhibition of ROS levels in human breast, colon, gastric, prostate, and bone cancer cell lines [15,16,17,18,19]. Conversely, a previous study has shown that normal human breast epithelial cells are more resistant to DATS-induced apoptosis and prone to inhibition of ROS production [20]. Based on this background information, this current work aims to further validate the effectiveness of DATS in preventing B[a]P-induced cancer initiation. In this study, we evaluated the ability of DATS to attenuate the early precancerous activities of B[a]P through changes in proliferation, clonogenic formation, ROS formation, oxidative DNA damage, and the expression of metabolic, antioxidant, DNA damage, and DNA repair proteins.

## 2. Materials and Methods

### 2.1. The Cell Line, Chemicals, and Reagents

MCF-10A cells were acquired from the American Type Culture Collection (ATCC, Manassas, VA, USA). The diallyl trisulfide (DATS) (purity 99.2%, 200 mM stock) was purchased from LKT Laboratories (St. Paul, MN, USA). DMSO, benzo[a]pyrene (B[a]P) (10 mM stock), Pierce BCA Protein Assay kit, and all other chemicals were obtained from ThermoFisher Scientific (Wilmington, DE, USA) and stored at −20 °C. DMEM/F-12 phenol red-free media, Hanks Balanced Salt Solution (HBSS), penicillin/streptomycin, epidermal growth factor (EGF), Phosphate Buffered Saline (PBS), Equine/horse serum (HS), and 10X trypsin-EDTA were obtained from Invitrogen (Carlsbad, CA, USA). Cholera toxin was obtained from Enzo Life Sciences (Plymouth Meeting, PA, USA). The CELLPRO-RO Roche Cell Viability and Proliferation Reagent WST-1 was purchased from Sigma-Aldrich (St. Louis, MO, USA). The Bromodeoxyuridine (BrdU) Cell Proliferation Assay kit was obtained from Cell Signaling Technology (Dancers, MA, USA). The Reactive Oxygen Species (ROS) Detection Assay Kit was obtained from BioVision Incorporated (Milpitas, CA, USA). The EpiQuik-8-OHdG DNA Damage Quantification Direct Kit (Colorimetric) was obtained from EpiGentek (Farmingdale, NY, USA). The Genomic DNA buffer kit, Qiagen Genomic-tip 20/G, and proteinase K were obtained from Qiagen (Germantown, MD, USA). Furthermore, 12–230 Separation Module, 8 × 25 capillary cartridges, 10× Sample buffer, Erk1 Primary Antibody, HeLa Lysate Controls, Anti-Rabbit Secondary Antibody, Streptavidin–HRP, and Luminol/Peroxide were obtained from ProteinSimple (San Jose, CA, USA). GAPDH was purchased from Cell Signaling (Danvers, MA, USA), and the primary antibody DNA polymerase β was obtained from Abcam (Boston, MA, USA).

### 2.2. Cell Culture

MCF-10A cells were cultured in DMEM/F12 phenol red-free media supplemented with EGFR (20 ng/mL), hydrocortisone (0.5 μg/mL), human insulin (10 μg/mL), horse serum (5%), cholera toxin (100 ng/mL), and 1% penicillin (100 U/mL)/streptomycin (0.1 mg/mL). Cells were grown to 75–90% confluence in a humidified incubator at 37 °C, 5% CO_2_. Media was changed every 3 days, and the cells were subcultured every 5 days.

### 2.3. Cell Treatments

MCF-10A cells were categorized into various treatment groups as follows: (1) DATS-treated (40, 60, or 80 μM), (2) B[a]P-treated (1 μM), or (3) B[a]P and DATS co-treatments (CoTx: 1 μM B[a]P and 40, 60, or 80 μM of DATS). For viability studies, cells were treated with or without DATS (12.5, 25, 50, 75, and 100 μM) and with or without B[a]P (0.01, 0.1, 0.25, 0.5, and 1 μM) for different periods (24 and 48 h). For clonogenic formation studies, cells were treated with 0.01, 1, and 2 μM B[a]P, or 1 μM B[a]P treated alone or concurrently (CoTx) with DATS (40, 60, or 80 μM) for 7 days. Untreated cells in media and 0.1% DMSO vehicle control were prepared for all treatments and experiments under low light conditions in serum-free media. Treated cells were incubated for 12, 24, or 48 h in a humidified incubator at 37 °C, 5% CO_2_.

### 2.4. Cell Harvesting

Following treatment, the adherent cells were harvested by trypsinization using trypsin-EDTA for 5–10 min in a humidified incubator at 37 °C, 5% CO_2_. After trypsinization, the cells were neutralized with media and centrifuged for 5 min at 1500 rpm. The supernatant was removed, and the cell pellets were suspended in PBS without Mg^2+^ or Ca^2+^.

### 2.5. Determination of Cell Viability

MCF-10A cells (2 × 10^4^/well) were plated in serum-free media into 84 wells of a flat bottom plate (100 μL/well) and adhered overnight in the incubator at 37 °C, 5% CO_2_. The media was then aspirated from each well and treated with 100 μL of media prepared with the above treatments three times at replicates of *n* = 8. Following the incubation period, cell viability was determined per the manufacturer’s instructions to the CELLPRO-RO Roche Cell Viability and Proliferation Reagent WST-1. The BioTek Synergy H1 Microplate Reader (Bio-Tek Instruments, Inc., Winooski, VT, USA) was used to measure absorbance at 440 nm.

### 2.6. Bromodeoxyuridine (BrdU) Cell Proliferation Assay

Cell proliferation was determined per the manufacturer’s instructions for the Cell Signaling Technology BrdU Cell Proliferation Assay Kit and previous studies [11]. MCF-10A cells (5 × 10^4^/well) were plated into 84 wells of a flat bottom plate (100 μL/well) for 12 and 24 h with treatments described above in triplicate at least three times at *n* = 8. Resuspended cells were incubated for BrdU incorporation for 24 h, followed by fixation, primary and secondary antibody labeling, and luminal enhancer solution. The BioTek Synergy H1 Microplate Reader (Bio-Tek Instruments, Inc., Winooski, VT, USA) was used to measure luminescence at 450 nm.

### 2.7. Clonogenic Formation Assay

Before assay, cells were cultured in 5% dextran-coated charcoal-treated HS-DMEM/F12 media with supplements (described above). Cells were seeded at a 2.5 × 10^2^/well density in a 6-well plate and allowed to adhere and proliferate for 7 days. The following week, the cells were covered in treated supplemented serum-free media with B[a]P (0.1, 1, and 2 μM), DATS (40, 60, and 80 μM), or DATS CoTx (40 μM DATS + 1 μM B[a]P) in replicates of *n* = 3 for an additional 7 days. Media was replaced every 5 days with appropriate treatment for two weeks. After two weeks, the media was aspirated, and the cells were fixed with glutaraldehyde solution for 30 min, dried overnight, washed, stained with crystal violet for 30 min, washed additionally, and dried overnight. The following day, the colonies were counted. The MCF-10A cells that were treated were compared to 0.1% DMSO vehicle control in serum-free media.

### 2.8. ROS Detection Assay

Resuspended MCF-10A cells (1 × 10^4^/well) were plated in serum-free media into 84 wells of a flat bottom plate (100 μL/well) and adhered overnight. In total, 100 μL of supplemented serum-free media was placed in each well with treatments described above at least three times at *n* = 8 for 12 and 24 h. The ROS determination protocol was followed according to the Reactive Oxygen Species (ROS) Detection Assay Kit from Bio Vision Incorporated (Milpitas, CA, USA) manufacturer’s instructions. 0.1% H_2_O_2_ was used as a positive control, and the BioTek Synergy H1 Microplate Reader (Bio-Tek Instruments, Inc., Winooski, VT, USA) was used to measure fluorescence at Ex/Em = 495/529.

### 2.9. 8-Hydroxy-2-Deoxyguanosine (8-OHdG) Detection

8-OHdG was measured using the EpiQuik 8-OHdG DNA Damage Quantification Direct Kit (Colorimetric), and the method used in prior studies [21] was followed.

### 2.10. Western Blotting

Cell pellets corresponded to untreated cells in media, 0.1% DMSO, B[a]P treated (1 μM), DATS treated (40 μM), and CoTx (1 μM B[a]P combined with 40 μM DATS) after 24 h treatment. 0.5% TritonX-100 was mixed with a protease inhibitor cocktail and combined with each pellet. The Pierce BCA Protein Assay kit was used to determine protein concentration. Each sample contained 50 μg of protein. 1:1000 dilution of primary antibody and secondary antibody were used. After the secondary antibody was incubated, the protein was identified, and the digital immunoblot was taken. The primary antibodies tested were CYP1A1 (ab235185) purchased from Abcam (Boston, MA, USA), Hypoxia Pathway Antibody Sampler Kit (#15792), AhR mAb (#83200), and loading control GAPDH mAb (#D16H11) purchased from Cell Signaling (Danvers, MA, USA).

### 2.11. ProteinSimple (Wes) Capillary Electrophoresis Western Analysis

Cell pellets corresponded to untreated cells in media, 0.1% DMSO vehicle control, B[a]P treated (1 μM), DATS treated (40 μM), and CoTx (1 μM B[a]P combined with 40 μM DATS) after 24 h treatment. 0.5% TritonX-100 was mixed with a protease inhibitor cocktail and combined with each pellet. The Pierce BCA Protein Assay kit was used to determine protein concentration. Each sample contained 2 mg/mL of protein used for Wes. 1:125 dilution of primary antibody and secondary antibody were used. The samples were prepared, heated at 95 °C for 5 min, and then loaded into the microplate. The Protein Standard Ladder, antibody diluent (blocking buffer), primary and secondary antibodies, Luminol/Peroxide, Streptavidin–HRP, and wash buffer were loaded into the corresponding microplate wells. Next, the microplate and capillary cartridge were placed into the ProteinSimple WES machine as per the manufacturer’s instructions (ProteinSimple, San Jose, CA, USA). After the capillary reaction was completed, the target protein was identified, and a digital immunoblot image was taken. The digital image of the blots was then quantified and analyzed using the ProteinSimple SW Compass 6.2.0 software program. ProteinSimple WES™ data were normalized using GAPDH. The primary antibodies tested were DNA polymerase β (ab26343), purchased from Abcam, and loading control GAPDH mAb (#D16H11), purchased from Cell Signaling.

### 2.12. Statistical Analysis

Each experiment was conducted in triplicate (*n* = 3) and averaged for three biological replicates. The data from all experiments were analyzed on GraphPad Prism 9.0 software (San Diego, CA, USA) using one-way analysis of variance (ANOVA) followed by Dunnett’s Multiple Comparison Test. The average values ± SEM display the results to determine statistically significant differences between the DMSO vehicle (*), B[a]P (#), and various treatment groups.

## 3. Results

### 3.1. The Effect of DATS and B[a]P on Cell Viability in MCF-10A Cells

**Figure 1:**
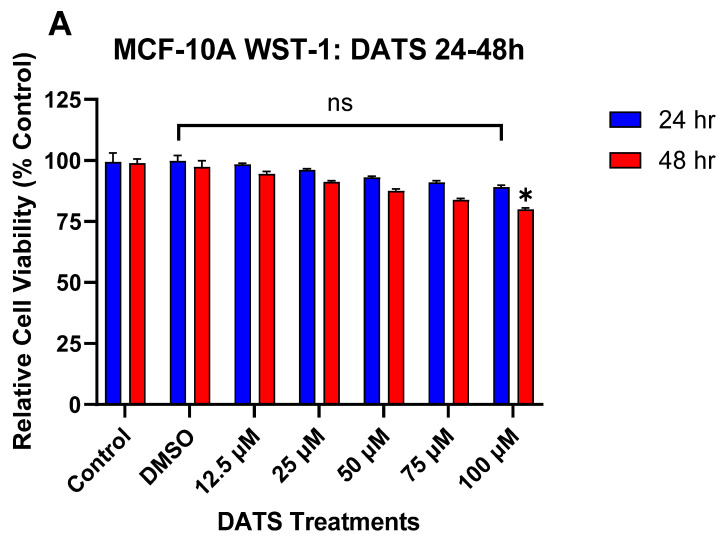

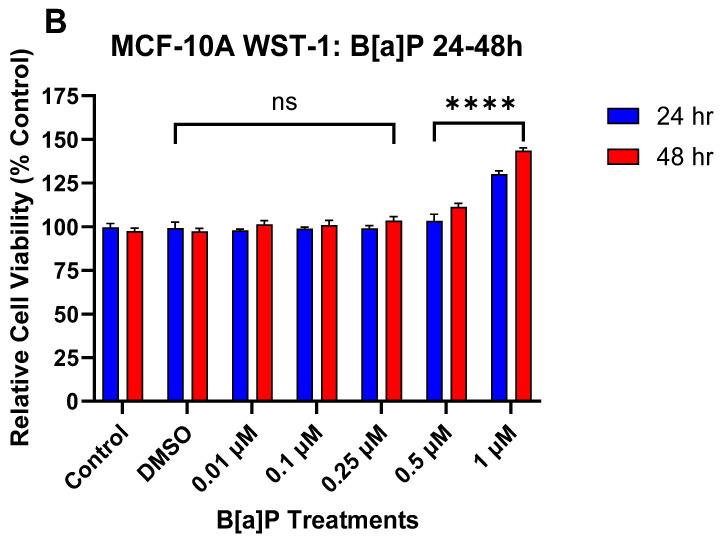
(**A**) The effect of DATS on the viability of MCF-10A cells. MCF-10A cells were treated with 0–100 μM DATS for 24 and 48 h. DATS had no significant effect between 12.5 and 75 μM. Treatment with 100 μM of DATS at 48 h significantly decreased cell viability compared to control. The graph displays all experiments conducted in *n* = 8 and averaged for three separate experiments. The data were assessed using one-way ANOVA and Dunnett’s multiple comparison test. The average values ± SEM display the results to determine significant differences between the vehicle control and treatments. (* *p* < 0.05 and ns indicates no significance). (**B**) The effect of B[a]P on viability of MCF-10A cells. MCF-10A cells were treated with 0.01–1 μM B[a]P for 24 and 48 h. Treatment with 1 μM B[a]P and above caused a significant increase in cell viability compared to the control. The graph displays all experiments conducted in *n* = 8 and averaged for three separate experiments. The data were assessed using one-way ANOVA and Dunnett’s multiple comparison test. The average values ± SEM display the results to determine significant differences between the vehicle control and treatments. (**** *p* < 0.0001 and ns indicates no significance).

The WST-1 assay was used to evaluate changes in cell viability of MCF-10A cells after treatment with B[a]P or DATS measured over 24 and 48 h periods. Cells treated with varying concentrations of DATS (12.5–75 μM) for both 24 and 48 h periods showed no significant effect on cell viability when compared to the control. Although a similar, non-significant effect was observed with the 100 μM DATS treatment at 24 h, the treatment with 100 μM DATS for 48 h caused a significant (*p* < 0.05) decrease in cell viability relative to the vehicle control (Figure 1A). Treatment with B[a]P concentrations equal to or higher than 0.5 μM significantly (*p* < 0.0001) increased cell viability over time compared to the vehicle control (Figure 1B). The most significant increase in viability, relative to the vehicle control, occurred at 1 μM of B[a]P treatment (Figure 1B). The cell viability data from both compounds were used to evaluate appropriate DATS or B[a]P concentrations for further studies.

### 3.2. DATS Inhibits B[a]P-Induced Cell Proliferation Using BrdU Proliferation Assay on MCF-10A Cells

**Figure 2:**
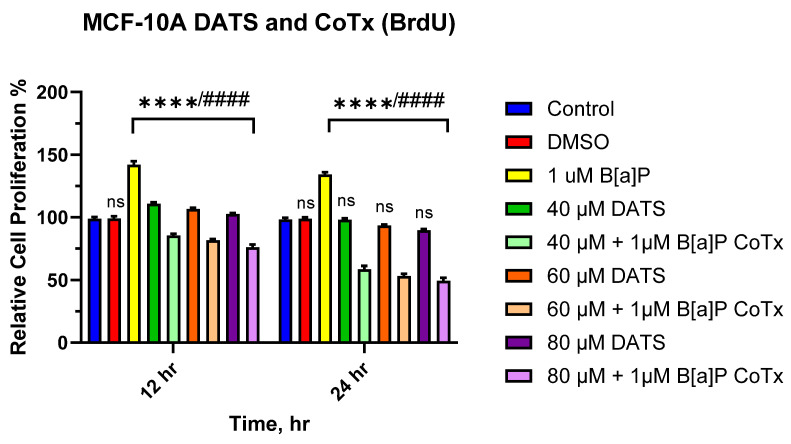
Cell proliferation percentage of MCF-10A cells treated with B[a]P and DATS. MCF-10A cells were treated with 1 μM B[a]P only, 40–80 μM DATS only, or 1 μM B[a]P + 40–80 μM CoTx for 12 and 24 h. The graph displays all experiments conducted in *n* = 8 and averaged for three separate experiments. The data were assessed using one-way ANOVA and Dunnett’s multiple comparison test. The average values ± SEM display the results to determine significant differences between DMSO vehicle, B[a]P, and various treatment groups. (ns indicates no significance, **** *p* < 0.0001 compared to the control, and #### *p* < 0.0001 when compared to B[a]P treatment).

The BrdU proliferation assay was used to further assess the effect of DATS and/or B[a]P on cell proliferation over time. BrdU was measured over a 12–24 h period. Exposure to 1 μM of B[a]P caused a significant increase in cell proliferation at 12 h (*p* < 0.0001) and 24 h (*p* < 0.0001) when compared to the vehicle control (Figure 2. Likewise, there was a significant (*p* < 0.0001) increase in cell proliferation following DATS (40, 60, and 80 μM) treatments when compared to both the vehicle control and a significant (*p* < 0.0001) decrease when compared to B[a]P alone at 12 h. However, cells treated with DATS (40, 60, and 80 μM) showed no significant effect when compared to the vehicle control at 24 h. Additionally, the CoTx (40–80 μM) significantly (*p* < 0.0001) inhibited cell proliferation when compared to 1 μM of B[a]P, respectively.

### 3.3. DATS Inhibits B[a]P-Induced Colony Formation in MCF-10A Cells

**Figure 3:**
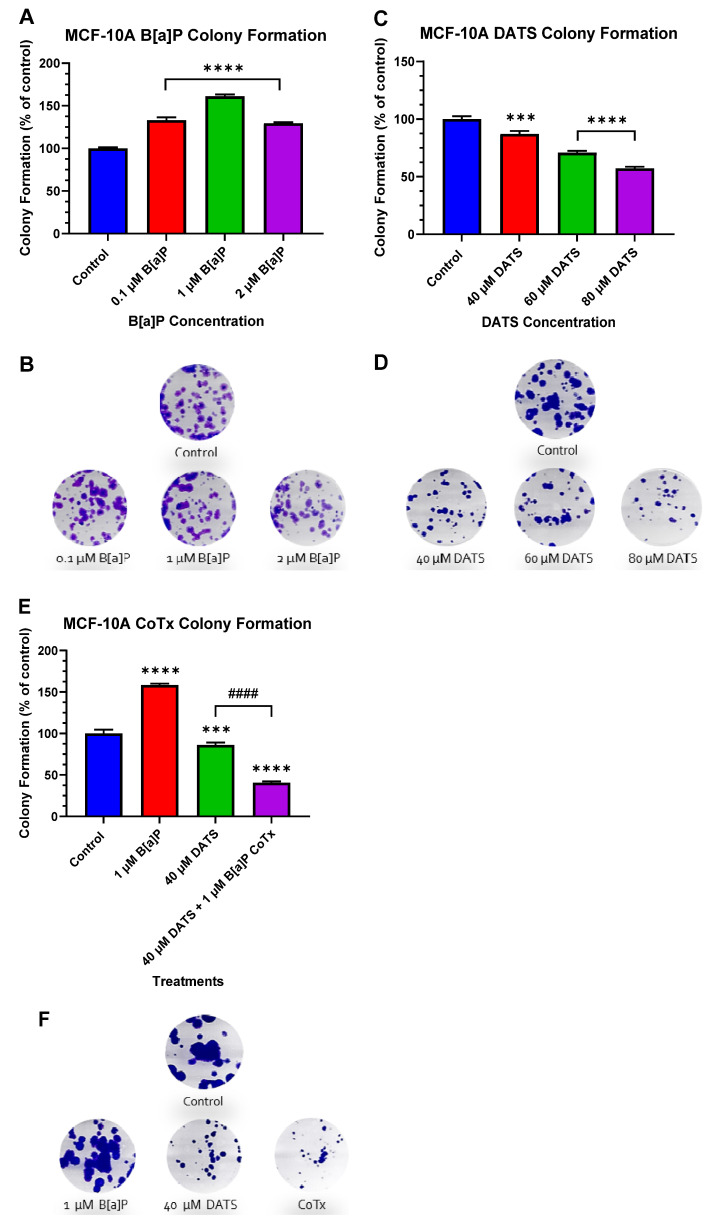
(**A**–**F**) Clonogenic formation of MCF-10A cells treated with B[a]P, DATS, and DATS CoTx. (**A**) Effects of B[a]P on colony formation of MCF-10A. (**C**) Effects of DATS on colony formation of MCF-10A cells. (**E**) Effects of 1 μM B[a]P alone, 40 μM DATS alone, and 40 μM CoTx on colony formation of MCF-10A cells. Cells were placed in phenol red-free DMEM supplement with 5% dextran-coated charcoal-treated HS for 24 h before plating. Then 250 cells/well were plated in six-well plates. Seven days later, cells were treated with 0.1% DMSO vehicle control. (**B**,**D**,**F**), and the graphs display all experiments conducted in *n* = 3 and averaged for three separate experiments. The data were assessed using one-way ANOVA and Dunnett’s multiple comparison test. The average values ± SEM display the results to determine significant differences between DMSO vehicle, B[a]P, and various treatment groups. (*** *p* < 0.001, **** *p* < 0.0001 compared to the control, and #### *p* < 0.0001 when compared to B[a]P treatment).

We used the clonogenic formation assay to examine the ability of a single adherent cell treated with B[a]P and/or DATS to survive over time and undergo clonogenic expansion (Figure 3A–F). The vehicle control exhibited the formation of a significant number of colonies. Compared to the vehicle control, treatment with B[a]P significantly (*p* < 0.0001) enhanced the number of colonies by 33%, 61%, and 29% for 0.1 μM, 1 μM, and 2 μM of B[a]P, respectively (Figure 3A,B). Cells treated with DATS exhibited a dose-dependent decrease in colony formation. Treatment with 40 (*p* < 0.001), 60 (*p* < 0.0001), and 80 (*p* < 0.0001) μM of DATS significantly decreased colony formation in a dose-dependent manner at 13%, 29%, and 43%, respectively, when compared to the vehicle control (Figure 3C,D). Additionally, we assessed the clonogenic formation of MCF-10A cells treated with 1 μM B[a]P alone, 40 μM DATS alone, or 40 μM CoTx. Treatment with 1 μM B[a]P alone significantly (*p* < 0.0001) increased the number of colony formations, reaching a maximum of approximately 58% above the control levels. Treatment with 40 μM DATS alone significantly (*p* < 0.001) decreased the number of colony formations by 14% compared to the vehicle control level and also significantly (*p* < 0.0001) reduced colony formation by 72% when compared to the 1 μM B[a]P. Treatment with 40 μM CoTx significantly (*p* < 0.0001) reduced the number of colony formations by 59% when compared to the control and by 118% when compared to the 1 μM B[a]P (Figure 3E,F). Furthermore, 40 μM CoTx led to a marked decrease in colony formation compared with 1 μM B[a]P and vehicle control alone.

### 3.4. DATS Suppresses the Accumulation of ROS in B[a]P-Treated MCF-10A Cells 

**Figure 4:**
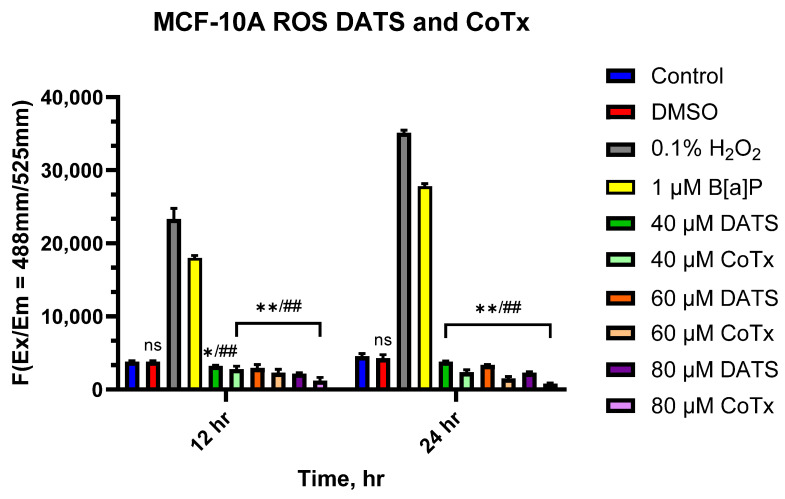
DATS inhibition of B[a]P-induced ROS in MCF-10A cells. The cells analyzed for ROS production were treated with B[a]P, DATS, or CoTx for 12 and 24 h. 0.1% Hydrogen peroxide was used as a positive control. The graphs display all experiments conducted in *n* = 3 and averaged for three separate experiments. The average values ± SEM display the results to determine significant differences between DMSO vehicle, B[a]P, and various treatment groups. (ns indicates no significance, * *p* < 0.05, ** *p* < 0.01 compared to the control, and ## *p* < 0.01 when compared to B[a]P treatment).

The levels of ROS resulting from MCF-10A cells treated with B[a]P and DATS were measured over 12 and 24 h periods (Figure 4). B[a]P caused a significant increase in ROS production, which peaked at 24 h; however, treatment with all DATS and CoTx (40–80 μM) concentrations exhibited a dose- and time-dependent response for all time points with an overall decrease in ROS production. MCF-10A cells treated with 40 μM DATS (*p* < 0.05) and 60–80 μM DATS (*p* < 0.01) indicated a decrease in ROS production by 30.9%, 33.8%, and 46.1% compared to the control, respectively. After 24 h, cells treated with 40–80 μM DATS (*p* < 0.01) also demonstrated reduced ROS production by 35.8%, 43.7%, and 82.1% compared to the control. Similarly, the 12 h CoTx had significantly (*p* < 0.01) suppressed B[a]P-induced ROS by 26.3%, 30%, and 43.6% at 40, 60, and 80 μM, respectively. For 24 h, CoTx (*p* < 0.01)) significantly suppressed B[a]P-induced ROS by 42.1%, 67.4%, and 128.7%, respectively, at 40, 60, and 80 μM. All treatments also indicated a significant (*p* < 0.01) decrease in ROS production when compared to the 1 μM B[a]P. As detected by ROS production, these results indicate treatments with DATS and CoTx effectively inhibited ROS formation.

### 3.5. DATS Inhibits B[a]P-Induced Oxidative (8-OHdG) DNA Damage in MCF-10A Cells

**Figure 5:**
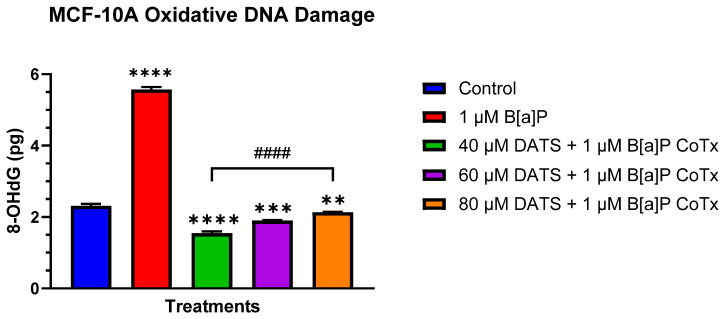
DNA damage detection of MCF-10A cells treated with DATS and/or B[a]P. MCF-10A cells were treated with 1 μM B[a]P only or 1 μM B[a]P + 40–80 μM CoTx for 24 h. The graph displays oxidative DNA damage indicated by 8-OHdG in pg. All experiments were conducted in *n* = 3 and averaged for three separate experiments. The data were assessed using an unpaired *t*-test. The average values ± SEM display the results to determine significant differences between DMSO vehicle, B[a]P, and various treatment groups. (** *p* < 0.01, *** *p* < 0.001, **** *p* < 0.0001 compared to the control, and #### *p* < 0.0001 when compared to B[a]P treatment).

The Epiquik 8-OHdG DNA Damage Quantification Direct Kit was used to measure the oxidative DNA damage (Figure 5). B[a]P (1 μM) significantly (*p* < 0.0001) increased the generation of 8-OHdG when compared to the vehicle control as a result of increased oxidative DNA damage. While the 40, 60, and 80 μM CoTx significantly (*p* < 0.0001) decreased 8-OHdG when compared to the 1 μM B[a]P, all CoTxs also significantly (40 μM CoTx *p* < 0.0001, 60 μM CoTx *p* < 0.001, and 80 μM CoTx *p* < 0.01) decreased 8-OHdG when compared to the control, respectively. While the CoTx for all concentrations of DATS caused a significant decrease in 8-OHdG formation, as an indicator of a reduction in oxidative stress and DNA damage, when compared to B[a]P alone, the 8-OHdG levels significantly increased to the control level with increasing concentrations of DATS in a dose-dependent manner.

### 3.6. Decrease in Aryl Hydrocarbon Receptor (AhR) Protein Expression by DATS in B[a]P-Treated MCF-10A Cells

**Figure 6:**
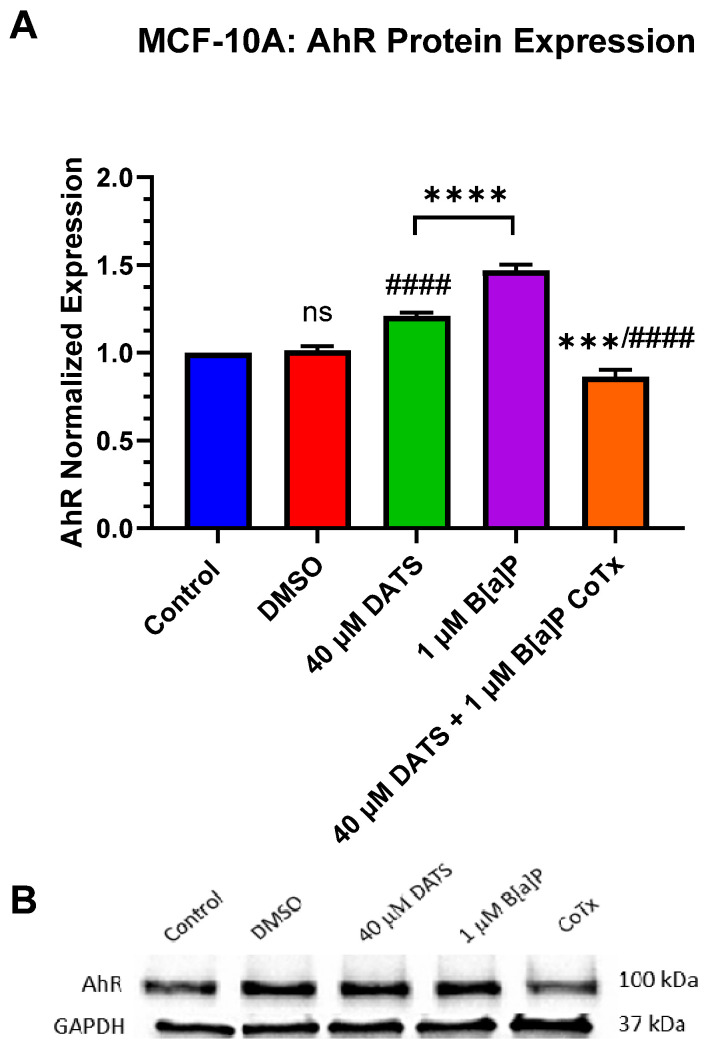
DATS and/or B[a]P effect on AhR expression in MCF-10A cells. Protein expression of AhR was measured by densitometry and normalized (**A**). The immunoblots represented the protein expression after 24 h post-treatment for AhR (**B**). The graphs display all experiments conducted in *n* = 3 and averaged for three separate experiments. The average values ± SEM display the results to determine significant differences between DMSO vehicle, B[a]P, and treatment groups. (ns indicates no significance, *** *p* < 0.001, **** *p* < 0.0001 compared to the control, and #### *p* < 0.0001 when compared to B[a]P treatment).

The inactive cytoplasmic aryl hydrocarbon receptor (AhR) is present in MCF-10A cells. AhR protein expression was evaluated after 24 h exposure to 1 μM B[a]P, 40 μM DATS, and 40 μM CoTx. A GAPDH loading control was used to normalize the protein expression of all treatments. All treatments were compared to the vehicle control (Figure 6A,B). Exposure to 1 μM B[a]P (*p* < 0.0001) significantly increased AhR expression when compared to the control alone (Figure 6A,B). Exposure to 40 μM DATS also significantly (*p* < 0.0001) increased AhR expression when compared to the control but to lower levels compared to 1 μM B[a]P. The 40 μM CoTx significantly (*p* < 0.0001) decreased AhR expression compared to the 1 μM B[a]P. Interestingly, the reduction of AhR expression by 40 μM CoTx was much more prominent than all treatments compared to B[a]P and the control. The presence of AhR expression was validated in all treatments using Western blot.

### 3.7. Decrease in Hypoxia-Inducible Factor-1beta/Aryl Hydrocarbon Receptor Nuclear Translocator (HIF-1β/ARNT) Protein Expression by DATS in B[a]P-Treated MCF-10A Cells

**Figure 7:**
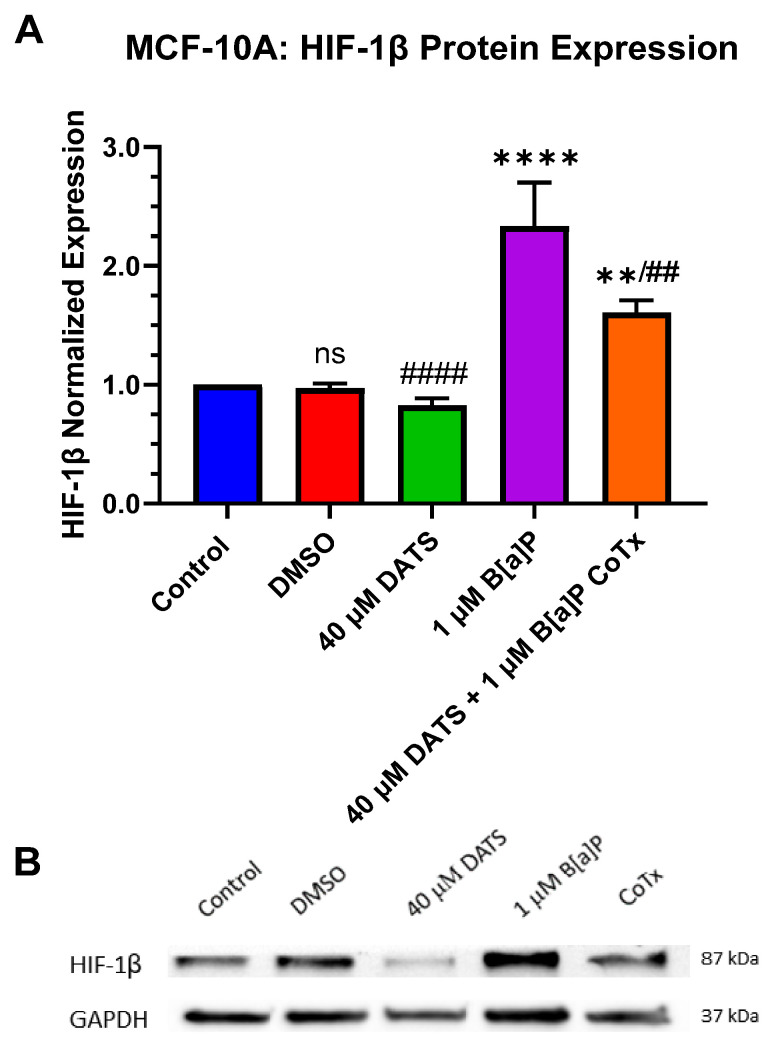
DATS and/or B[a]P effect on HIF-1β expression in MCF-10A. Protein expression of HIF-1β was measured by densitometry and normalized (**A**). The immunoblots represented the protein expression after 24 h post-treatment for HIF-1β (**B**). The graphs display all experiments conducted in *n* = 3 and averaged for three separate experiments. The average values ± SEM display the results to determine significant differences between DMSO vehicle, B[a]P, and treatment groups. (ns indicates no significance, ** *p* < 0.01, **** *p* < 0.0001 compared to the control, and ## *p* < 0.01, #### *p* < 0.0001 when compared to B[a]P treatment).

B[a]P produces a hypoxic response in B[a]P-treated MCF-10A cells. The hypoxia-inducible factor-1beta (HIF-1β/ARNT) can be induced to cause upregulation in the hypoxic response. HIF-1β protein expression was evaluated when treated with 1 μM B[a]P, 40 μM DATS, and 40 μM CoTx. Protein expression was measured using Western blot analysis. A GAPDH loading control was used to normalize the protein expression of all treatments. All treatments were compared to the vehicle control and the 1 μM B[a]P treatment (Figure 7A,B). Exposure to 1 μM B[a]P significantly (*p* < 0.0001) increased HIF-1β expression when compared to the control. The 40 μM DATS alone treatment significantly (*p* < 0.0001) decreased HIF-1β expression and 40 μM CoTx also significantly (*p* < 0.01) decreased HIF-1β expression when compared to 1 μM B[a]P alone, respectively.

### 3.8. Decrease in Cytochrome P450 1A1 (CYP1A1) Protein Expression by DATS in B[a]P-Treated MCF-10A Cells

**Figure 8:**
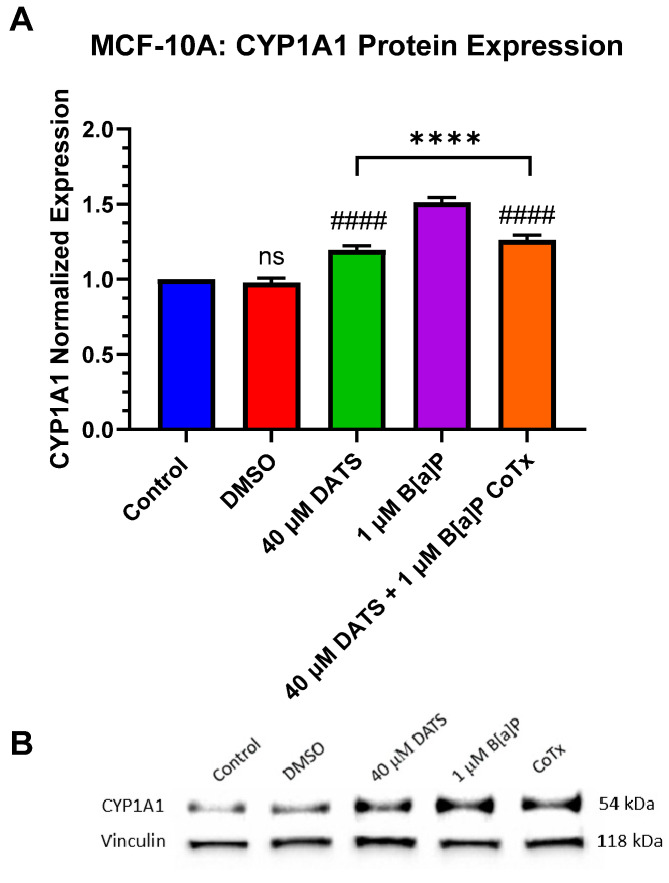
DATS and/or B[a]P effect on CYP1A1 expression in MCF-10A cells. Protein expression of CYP1A1 was measured by densitometry and normalized (**A**). The immunoblots represented the protein expression after 24 h post-treatment for CYP1A1 (**B**). The graphs display all experiments conducted in *n* = 3 and averaged for three separate experiments. The average values ± SEM display the results to determine significant differences between DMSO vehicle, B[a]P, and treatment groups. (ns indicates no significance, **** *p* < 0.0001 compared to the control and #### *p* < 0.0001 when compared to B[a]P treatment).

Since cytochrome P450 1A1 (CYP1A1) expression is present in MCF-10A cells, CYP1A1 protein expression was evaluated when treated with 1 μM B[a]P, 40 μM DATS, and 40 μM CoTx. A GAPDH loading control was used to normalize the protein expression of all treatments. All treatments were compared to the vehicle control (Figure 8A,B). Exposure to 40 μM DATS alone and 40 μM CoTx significantly (*p* < 0.0001) increased CYP1A1 expression when compared to the control and conversely, significantly (*p* < 0.0001) decreased CYP1A1 expression when compared to the 1 μM B[a]P. Exposure to 1 μM B[a]P (*p* < 0.0001) increased CYP1A1 expression when compared to the control alone, respectively (Figure 8A,B). The presence of CYP1A1 expression was validated in all treatments through Western blot.

### 3.9. Induction of DNA Polymerase Beta (POLβ) Protein Expression by DATS in B[a]P-Treated MCF-10A Cells

**Figure 9:**
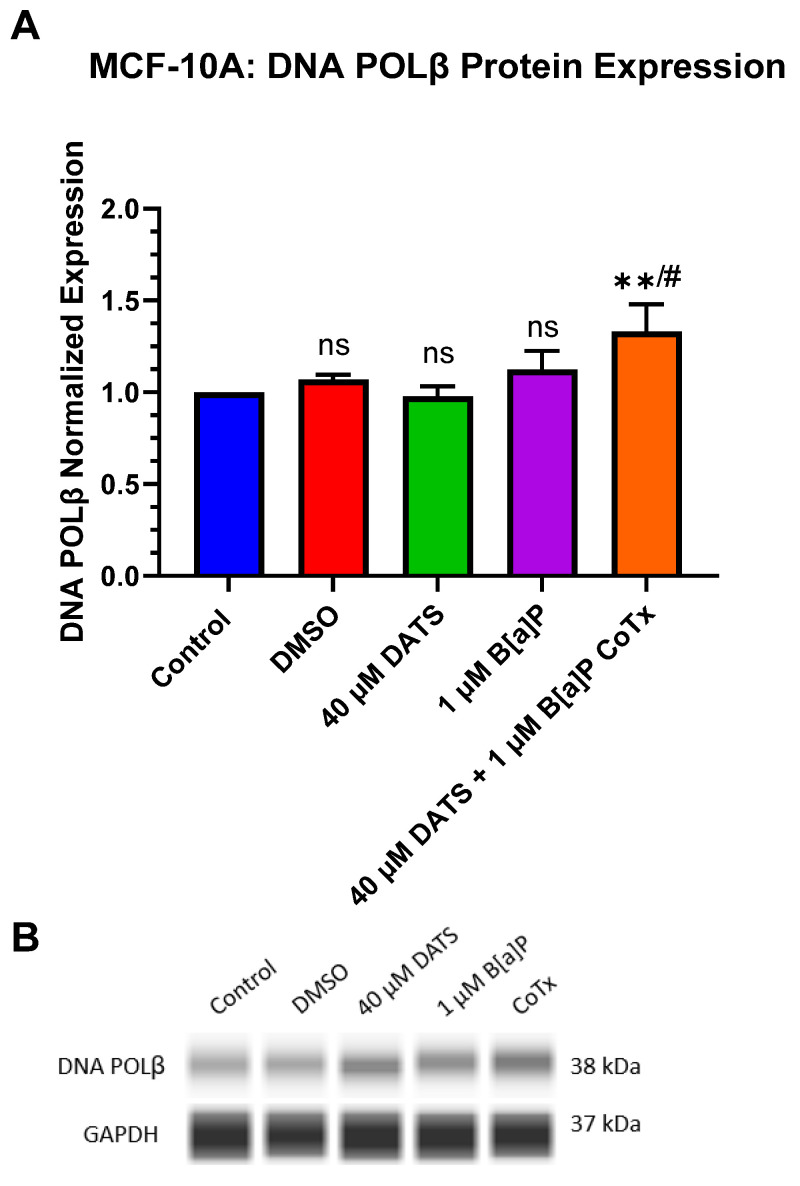
DATS and/or B[a]P effect on DNA POLβ expression in MCF-10A. Normalized DNA POLβ protein expression was measured by the ProteinSimple SW Compass software for each of the five samples, one of which is a control, and the others are treated with DMSO, DATS, B[a]P, and CoTx (**A**). Immunoblots from the ProteinSimple SW Compass 6.2.0 software corresponded to the protein expression for the same sample treatments (**B**). The graphs display all experiments conducted in *n* = 3 and averaged for three separate experiments. The average values ± SEM display the results to determine significant differences between DMSO vehicle, B[a]P, and treatment groups. (ns indicates no significance, ** *p* < 0.01 compared to the control, and # *p* < 0.05 when compared to B[a]P treatment).

POLβ protein expression was evaluated when treated with 1 μM B[a]P, 40 μM DATS, and 40 μM CoTx. The lower concentration of 40 μM CoTx was utilized due to the increase in oxidative DNA damage with increasing concentrations of DATS CoTx. A GAPDH loading control was used to normalize the protein expression of all treatments. All treatments were compared to the control and the 1 μM B[a]P treatment (Figure 9A,B). Exposure to 40 μM CoTx significantly (*p* < 0.01) increased POLβ expression when compared to the control, and significantly (*p* < 0.05) increased POLβ expression when compared to 1 μM B[a]P alone, respectively (Figure 9A,B).

## 4. Discussion

The polycyclic aromatic hydrocarbon (PAH), B[a]P, is formed during the incomplete combustion of organic materials [10]. In vitro and in vivo studies have shown the carcinogenic effect resulting from exposure to B[a]P and its reactive metabolites, thus producing mutagenesis via the formation of ROS-induced oxidative damage, DNA strand breaks, DNA adducts, mutations, and tumorigenesis leading to the development of cancer [11,13]. Natural remedies have been used for centuries to cure human diseases and illnesses. Garlic OSCs, well-known phytochemicals, were shown in epidemiological, preclinical, and clinical studies to have a number of protective biological properties, including antioxidant, cancer preventive, anticancer, and anti-inflammatory activities [22,23]. In an intervention study performed by Lawson et al. [24], human subjects were orally administered 7 g of crushed garlic or 64.4 mg (730 μM) of DATS on a daily basis. Based on these findings, daily treatments of 40 μM, 60 μM, and 80 μM of DATS used in our study are equivalent to daily consumption of 3.5 mg, 5.2 mg, and 7.07 mg in humans, respectively. Thus, the concentrations used in this study are well within tolerated physiological ranges in humans. Our lab and others have previously reported that DATS affects chemical-induced carcinogenesis through the modulation of aqueous peroxides, the induction of cell cycle arrest, and DNA damage in breast epithelial cells [13,21,22,25]. Thus, the focus of this study was to provide further evidence of DATS’ attenuation on B[a]P-induced precancerous activities through alterations in cell proliferation, clonogenic formation, ROS formation that can lead to DNA damage, and interplay between the various proteins expressed (AhR, HIF-1β, CYP1A1, and DNA POLβ) as indicators of DNA damage which may lead to mutations and neoplastic transformation of B[a]P-treated normal breast epithelial MCF-10A cells.

Our lab previously revealed that B[a]P induced cell proliferation, while treatment with B[a]P in combination with DATS did not alter cell proliferation in non-neoplastic MCF-10A breast epithelial cells [13]. In addition, several studies have concluded that normal cells are significantly more resistant to apoptosis induction by DATS than cancer cells [26,27] through an elusive selective mechanism [28]. In the work by Nkrumah-Elie et al. [13], DATS was shown to be an effective attenuator of B[a]P-induced effects by inhibiting lipid peroxide formation, DNA damage, and maintaining normal breast epithelial cells in G2/M and S-phase shifts after 24 h of DATS/B[a]P co-exposure thus exhibiting a protective mechanism. In our study, we found that DATS CoTx was effective in inhibiting B[a]P-induced cell proliferation at 12 h and 24 h, with a more pronounced effect at 24 h. DATS alone and CoTx were also found to significantly inhibit (*p* < 0.001 and *p* < 0.0001) clonogenic formation after 7 days of treatments, whereas B[a]P significantly induced (*p* < 0.0001) clonogenic formation during the same time point. Therefore, DATS is shown to be an effective attenuator of B[a]P-induced cell proliferation. Our results may validate previous findings that DATS may attenuate the proliferative effects of procarcinogens such as B[a]P and its metabolites [28].

The environmental pollutant B[a]P is a recognized reproductive and developmental toxicant [29]. The main mechanistic action of B[a]P is through direct damage to DNA by forming DNA adducts, leading to the development of DNA lesions and mutations [30,31]. In this study, B[a]P significantly induced (*p* < 0.0001) ROS generation, which is a possible precursor for oxidative damage. At the cellular level, ROS are short-lived, highly reactive, and oxygen-containing molecules that may induce DNA damage and affect DNA damage response [32]. Through research previously published by our group and those presented in this study, DATS was shown to attenuate ROS in the form of lipid peroxides in normal cells and effectively inhibit free radical induction induced by carcinogens [13].

Our lab and others have shown that B[a]P-induced oxidative stress may lead to DNA strand breaks in primary breast epithelial cells and human breast milk cells [21,33,34,35]. In Nkrumah-Elie et al.’s work [13], 24 h of exposure to DATS was shown to inhibit cellular carcinogenesis processes initiated by B[a]P-induced exposure in human breast epithelial MCF-10A cells, including DNA strand break formation, lipid peroxide production, and cell cycle arrest, thus being an effective attenuator of early carcinogenic activity and genotoxicity. Exclusive to the research performed in our lab, the literature has not shown a significant assessment of OSCs’ effect on inhibiting DNA strand breaks through the activation of DNA repair. The DNA repair system maintains genomic integrity via five major pathways, namely, base excision repair (BER), nucleotide excision repair (NER), mismatch repair (MMR), non-homologous end-joining (NHEJ), and homologous recombination (HR) [36,37]. BER is a conserved mechanism that involves oxidative damage, natural hydrolysis, and alkylation. The development of mutations results from the incorrect pairing of substituting 8-oxo-G with adenine [38]. Here, we demonstrated that B[a]P significantly induced oxidative DNA damage through a significant increase (*p* < 0.0001) in 8-oHdG at a concentration of 1 μM in normal breast cells. Studies have shown that the development of DNA adducts and oxidative lesions can be caused by exposure to PAHs such as B[a]P [39]. Our findings indicate that 1 μM of B[a]P co-treated with varying concentrations of DATS ranging from 40 to 80 μM significantly attenuated B[a]P-induced increases in 8-oHdG levels in normal breast epithelial cells, indicating a reduction in oxidative DNA damage and stress. In this study, 40 μM CoTx was the most effective concentration in inhibiting B[a]P-induced DNA damage. These results, coupled with the decrease in cell proliferation and ROS formation, support data in our previous studies that DATS induced a reduction in oxidative stress, thus leading to oxidative DNA damage.

The aryl hydrocarbon receptor (AhR) pathway integrates xenobiotic metabolism and development. AhR and the cytoplasmic receptor are activated by DNA response elements in response to endogenous and environmental signals to control gene expression [40]. AhR forms a heterodimer complex (AhR/ARNT) by binding to the xenobiotic responsive elements (XRE), where transcription is initiated in conjunction with the hypoxia-responsive elements (HRE) [40]. B[a]P is a main ligand of AhR that directly binds to the receptor and induces its biological effects associated with carcinogenesis in cancer and the development and progression of breast cancer [41,42]. In this study, AhR expression was significantly (*p* < 0.0001) induced following 24 h exposure to 1 μM B[a]P. The CoTx significantly decreased (*p* < 0.0001) the AhR response at the same time point, thus attenuating the AhR expression in B[a]P-treated breast epithelial cells.

ARNT/HIF-1β plays a key role in responding to oxygen deprivation (hypoxia) or microenvironmental stressors [40]. Here, we demonstrated that ARNT/HIF-1β expression was significantly (*p* < 0.0001) induced following 24 h exposure to 1 μM B[a]P. CoTx significantly decreased (*p* < 0.01) ARNT/HIF-1β response at the same time point in B[a]P-treated MCF-10A cells. In the work by Uno et al. [43], 100 μM DATS CoTx concurrently combined with 1 μM B[a]P enhanced B[a]P-induced AhR recruitment and histone acetylation on CYP1A1 in HepG2 liver cancer cell line. The findings of Uno et al. [43] support our findings in that higher concentrations of 100 to 200 μM DATS will induce cell death and oxidative stress. Uno et al. further demonstrate that these high doses of DATS increase AhR recruitment induced by B[a]P on the CYP1A1 gene. Our data shows that lower doses of 40 μM DATS CoTx significantly decreased AhR (*p* < 0.0001) and ARNT/HIF-1β (*p* < 0.01) with no impact on cell death and reduced ROS when compared to 1 μM B[a]P alone in the non-cancerous MCF-10A cells. These results, coupled with the decrease in AhR expression by DATS, suggest that OSCs may further inhibit transcription at the intracellular level by reducing the binding and activation of HIFs from B[a]P-induced toxicity.

The expression of cytochrome P450 enzymes, such as CYP1A1, is a signature hallmark of AhR signal transmission activation and metabolism [41]. CYP1A1 is a major enzyme that plays a role in carcinogenesis and tumor progression by inducing AhR by binding environmental pollutants such as B[a]P and inhaling chemicals that mitigate their biological and toxic effects [44]. In the current study, CYP1A1 protein expression was significantly (*p* < 0.0001) induced along with an increase in AhR and ARNT/HIF-1β protein expression following 24 h exposure to 1 μM B[a]P. These results correlate well with previous studies demonstrating the protein expression of CYP1A1, CYP1B1, ARNT/HIF-1β, and AhR receptor proteins in mammary epithelial cells following 24 h exposure to polycyclic aromatic hydrocarbons [45]. CYP1A1 expression was significantly increased (*p* < 0.0001) in MCF-10A cells treated with DATS alone or DATS CoTx when compared to the control. However, when compared to B[a]P alone, CYP1A1 protein expression was significantly reduced (*p* < 0.0001) in DATS or DATS CoTx samples. These findings, coupled with the decreased expression of AhR and ARNT/HIF-1β in CoTx samples, indicate that while single DATS exposure induces CYP1A1 expression in mammary epithelial cells, DATS CoTx attenuates the B[a]P-induced expression of AhR, ARNT/HIF-1β, and CYP1A1. These results correlate well with previous studies that have shown natural products may exert their chemopreventive effects by increasing or inhibiting CYP1A1 expression [46,47]. More specifically, co-treating HEPG2 hepatoma cells with 1 μM B[a]P and varying concentrations (50–100 μM) of DATS was found to induce CYP1A1 expression in a recent study [43], while an earlier study demonstrated that CYP1A1 expression was inhibited in HEPG2 microsomes when treated with 4 μM B[a]P in combination with 10 or 100 μM DATS [48]. Based on our previous and current findings [45,49,50,51,52], the expression of AhR, HIF-1β, and CYP1A1 in combination with decreased 8-oHdG and ROS levels after exposure to the DATS CoTx suggest that natural products like OSCs may exert their chemopreventive effect by inhibiting DNA adduct formation and competing with PAHs for both AhR and HIF-1β receptors leading to the attenuation of CYP1A1 protein expression thus attenuating B[a]P-induced toxicity in mammary epithelial cells. These studies suggest that DATS may play a role in protection from the toxicological effects of environmental contaminants such as B[a]P.

The X family of DNA polymerases (POLX) are key enzymes required during processes such as DNA replication, repair, synthesis, recombination, cell cycle control, and DNA damage checkpoint control [53]. DNA polymerase β (POLβ) is a DNA repair enzyme whose major function is contributed to BER by POLβ filling in a single nucleotide gap on an apurinic/apyrimidinic site to repair lesion damage from ROS and alkylating agents [54]. The experiments conducted in this study demonstrated that B[a]P had no effect while the garlic OSC, DATS, significantly increased POLβ protein expression when compared to the control. In this study, 40 μM DATS CoTx significantly (*p* < 0.01) increased POLβ response following 24 h exposure. To our knowledge, no study has reported a relationship between DATS and DNA repair enzymes such as POLβ. The induction of POLβ with 40 μM CoTx also correlates with the decrease in ROS formation and 8-oHdG levels in MCF-10A cells treated with the same concentration of DATS. These findings implicate POLβ as a key player in protecting normal cells from the genetic instability and genotoxicity produced by environmental toxicants such as B[a]P. Our lab’s future studies will include a more in-depth study concerning the role POLβ⁠ may play in DATS’s protection from the genotoxicity and genetic instability caused by B[a]P. We will also further investigate DATS’s role in the inhibition of cancer transformation using additional cell lines. These future studies will provide more insight into garlic’s cytoprotective role in DNA repair for breast cancer prevention.

## 5. Conclusions

**Figure 10:**
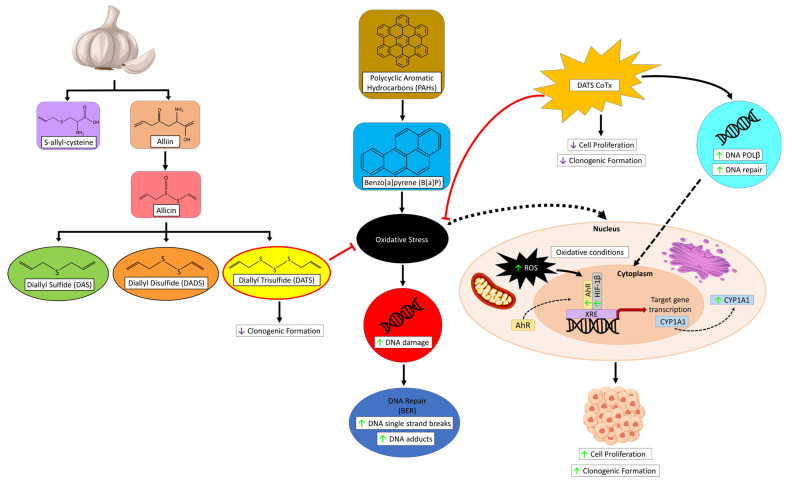
Schematic diagram and graphical abstract for our proposed mechanism of DATS in B[a]P-induced normal breast epithelial MCF-10A cells. The green arrow indicates increase, and the purple arrow indicates decrease. DATS can inhibit clonogenic formation. Whereas DATS CoTx can upregulate DNA repair and DNA POLβ, conversely inhibiting cell proliferation, clonogenic formation, and oxidative stress in the form of ROS. B[a]P can induce oxidative stress, thus upregulating ROS formation, AhR, HIF-1β, and CYP1A1, leading to an increase in DNA damage in the form of DNA single-strand breaks and DNA adducts, increasing cell proliferation and clonogenic formation.

These results suggest that DATS inhibits activities related to early-stage initiation of carcinogenesis, including alterations in cell proliferation, clonogenic formation, ROS formation that can lead to DNA damage, and the expression of various proteins (AhR, HIF-1β, and CYP1A1) while increasing the expression of the DNA repair protein DNA POLβ in MCF-10A cells (Figure 10). Our results show that B[a]P induces oxidative stress, thereby leading to cancer initiation through the development of GC: TA mispairing mutations and DNA adducts, while the CoTx may prevent cancer initiation through the attenuation of ROS formation and prevent the generation of GC: TA transversion mutations in MCF-10A cells. In this study, DATS was effective in the attenuation of early-stage B[a]P-induced carcinogenesis in non-cancerous breast epithelial MCF-10A cells. Therefore, garlic is believed to be an effective chemopreventive agent due to its antioxidant and antitumor abilities. Further research is needed to fully elucidate the mechanism of DATS and the findings obtained from our results.

## Data Availability

Data are contained within the article.
